# Multi-Label Feature Selection with Feature–Label Subgraph Association and Graph Representation Learning

**DOI:** 10.3390/e26110992

**Published:** 2024-11-18

**Authors:** Jinghou Ruan, Mingwei Wang, Deqing Liu, Maolin Chen, Xianjun Gao

**Affiliations:** 1 School of Computer Science, Hubei University of Technology, Wuhan 430068, China; jinghouruan@hbut.edu.cn (J.R.); 2010311212@hbut.edu.cn (D.L.); 2 School of Smart City, Chongqing Jiaotong University, Chongqing 400074, China; maolinchen@whu.edu.cn; 3 School of Geosciences, Yangtze University, Wuhan 430100, China; junxgao@yangtzeu.edu.cn

**Keywords:** multi-label data, feature selection, feature–label subgraph association, graph representation learning, optimal feature subset

## Abstract

In multi-label data, a sample is associated with multiple labels at the same time, and the computational complexity is manifested in the high-dimensional feature space as well as the interdependence and unbalanced distribution of labels, which leads to challenges regarding feature selection. As a result, a multi-label feature selection method based on feature–label subgraph association with graph representation learning (SAGRL) is proposed to represent the complex correlations of features and labels, especially the relationships between features and labels. Specifically, features and labels are mapped to nodes in the graph structure, and the connections between nodes are established to form feature and label sets, respectively, which increase intra-class correlation and decrease inter-class correlation. Further, feature–label subgraphs are constructed by feature and label sets to provide abundant feature combinations. The relationship between each subgraph is adjusted by graph representation learning, the crucial features in different label sets are selected, and the optimal feature subset is obtained by ranking. Experimental studies on 11 datasets show the superior performance of the proposed method with six evaluation metrics over some state-of-the-art multi-label feature selection methods.

## 1. Introduction

When faced with complicated information presentation, single-label data are incompatible with real-world applications for multi-level and multi-view data processing. Multi-label data [1] are intended to express a sample belonging to one or more categories simultaneously and are generally applied in the fields of text [2,3], audio [4,5], image [6,7], biology [8,9], and so on. In general, multi-label data involve high-dimensional feature space and many irrelevant and redundant features, which affects the classification accuracy and increases the computational complexity [10]. As a result, data dimensionality reduction has become a research hotspot with significant attention, which simplifies the feature space and highlights the correlation of features, helping to reveal the relationship between features and labels.

As one of the important approaches of data dimensionality reduction, feature selection [11] purports to select crucial features from the original data, which reduces the influence of irrelevant features and maintains the interpretability of feature subsets. The challenges of multi-label feature selection are the correlation of labels, the redundancy between features and the excessive dimensions of data. There are three categories based on the search strategy: wrapper, embedded and filter [12]. Among them, wrapper methods involve information interaction with an off-the-shelf classifier, in which the performance is used as a metric for evaluating the quality of feature subsets [13,14]. Embedded methods directly incorporate the process of selecting features as part of a classifier’s training, designing objective functions and adding constraint terms to emphasize the geometric structure [15]. For example, Jian et al. [16] proposed mapping label information to a low-dimensional space to capture label correlations and guide feature selection, minimizing the impact of imperfect label data (MIFS). Huang et al. [17] used manifold learning to transform the logical label space into a Euclidean space, constraining sample similarity with numerical labels (MCLS). Zhang et al. [18] used $l_{1}$ norm and $l_{2,1}$ norm regularization to identify label-specific and group-specific features, incorporating instance and label-group correlations to execute feature selection (GLFS). Zhang et al. [19] applied a low-dimensional embedding to capture local label correlations and co-occurrence relationships, ensuring convergence through $l_{2,1}$ norm regularization (MDFS). However, both wrapper and embedded methods require large space complexity as well as many hyperparameters and are dependent on classifiers, which may lead to overfitting [20]. Filter methods evaluate the feature importance by considering the distribution of features, which is independent of the classifier and requires relatively less running time [21,22]. It is categorized into two cases: one transforms multi-label data into single-label data, and another improves or directly proposes a new criterion. However, filter methods ignore the influence of the classifier, and the feature importance does not fully consider the correlation of labels.

Traditional multi-label feature selection methods are incompatible with analyzing the correlation of features, making it difficult to extract the global information in multi-label data. To compensate for this issue, information theory [23] was introduced to calculate the correlation through the ideas such as statistical analysis, entropy and mutual information [24,25,26]. Moreover, the relationship between features and labels is quantified to some extent [27], and features with strong label dependency are selected based on evaluation metrics and ranking. However, the relationship between features and labels is usually based on a single evaluation metric, which makes it difficult to consider the correlation of labels and fails to grasp the purpose of multi-label classification tasks.

From the perspective of the label, the key issue of feature selection lies in a complete and accurate understanding of the relationship between features and labels. As a heuristic search strategy, evolutionary computation [28] uses an inductive algorithm as a “black box” without any prior knowledge to discover possible crucial features [29], and the co-occurrence pattern of labels is comprehensively described by an objective. Considering that the multi-label data involve multiple objectives, multi-objective optimization [30,31,32] is used to balance these objectives, such as improving classification accuracy and reducing the number of selected features, and the feature subsets are obtained that are outstanding in a variety of aspects. However, the Pareto front [33,34] in multi-objective optimization still exhibits obvious feature redundancy for specific labels, making the effectiveness of data dimensionality reduction incomplete.

To obtain the optimal feature subset, it is necessary to explore the relationship between features and labels. Among them, graph theory [35,36] is commonly used to show the connection between objects, where the nodes usually represent the entities in multi-label data (e.g., feature or label), while edges represent the associations between nodes (e.g., relationship or correlation), and they are reflected by the connection of nodes and edges. Furthermore, the relationship between features and labels is intensively analyzed to reduce the data dimensionality more effectively. However, each label acts as an independent objective, and the connection of nodes grows exponentially with the increase in label dimensions, which affects the efficiency of feature selection.

This paper proposes a multi-label feature selection method based on feature–label subgraph association with graph representation learning (SAGRL). Among them, features and labels are mapped as nodes in the graph structure, and highly relevant nodes are aggregated into the same class to form feature and label sets. They are combined to construct feature–label subgraphs, which provide sufficient solution space for feature selection. Furthermore, features in each subgraph are ranked among multiple objectives from the perspective of labels to replace the previous feature set. For the feature and label nodes in each new subgraph, feature combinations are extracted by graph representation learning. The selected features are ranked to determine their importance and obtain the optimal feature subset, making them more representative. The main contributions of the paper are as follows:

- An SAGRL is proposed for multi-label feature selection, which considers features and labels as nodes to construct feature–label subgraphs, and the relationship is adjusted in each subgraph.
- Features and labels are mapped into the graph structure, which are aggregated to mine the intrinsic structural information of multi-label data and highlight the correlation of features or labels.
- The subgraph association is updated based on multi-objective optimization to choose the Pareto front feature set, remove redundant features from the original data, and reconstruct new subgraphs.
- The subgraphs are combined with augmentation paths to match feature and label sets, and the optimal feature subset is obtained by analyzing and ranking the importance of nodes.

The remainder of this paper is structured as follows. In Section 2, the related work is briefly reviewed. In Section 3, the proposed method involves concepts that are briefly introduced. In Section 4, the proposed method is described in detail. Experimental results are presented and discussed in different aspects in Section 5. Finally, conclusions and future work are presented in Section 6.

## 2. Related Work

In multi-label feature selection, information theory is mainly used to measure the correlation of entities (features or labels). Evolutionary computation is mainly used as a way to solve combinatorial optimization problems. The graph structure is mainly used to construct the relationship between entities. In this section, a brief review of related work is presented in the following subsection.

### 2.1. Multi-Label Feature Selection Method Based on Information Theory

Information theory evaluates the feature importance by quantifying the relationship between features and labels. Doquire et al. [37] employed PPT to transform the problem and a greedy MI-based search algorithm to capture dependencies between labels and features (PPT-MI). Read et al. [38] explained that PPT focuses on capturing relationships between labels while pruning and reducing overfitting (PPT-CHI). Zhang et al. [39] made two assumptions about the probability distribution of labels to consider the theoretical basis, the joint mutual information was utilized to approximate the higher-order mutual information, and the interaction weights were designed to distinguish the correlation of labels. Lee et al. [40] considered label interactions when evaluating feature dependency and selected an effective feature subset by maximizing the dependency between the selected features and labels (PMU). Li et al. [41] trained classifiers for the information granulation of each label, which selected features with the most relevant granularity to labels and the least redundancy in the feature subset. Hu et al. [42] proposed a relevance term on weight for focusing on the ratio of change in the amount of undetermined and determined information, which was fused with the evaluation of feature relevance. Lee et al. [43] calculated 2-degree interactions and a part of 3-degree interactions among features and labels to evaluate the dependency of input features in multivariate situations (D2F). Gonzalez-Lopez et al. [44] proposed two distributed methods for vectorized forms between feature and label sets, and the feature subset was obtained by maximizing the number of the $l_{2}$ norm and geometric mean of mutual information measures. However, information theory is based on the static probability distribution and lacks the ability to dynamically adjust the feature subset, which is insufficient to support different types of data, and it also fails to consider label co-occurrence well.

### 2.2. Multi-Label Feature Selection Method Based on Evolutionary Computation

Evolutionary computation gradually finds the optimal feature subsets by iteratively changing the feature combinations and assessing its contribution by the evaluation metrics. Hancer et al. [45] proposed an efficient pre-elimination process, an enhanced initialization scheme, and an exploration phase inspired by genetic operators, and a selection strategy based on standard statistical measures and special congestion distances was designed to consider the properties of feature combinations. Karagoz et al. [28] extracted a subset of non-dominated features by means of multi-label classification tasks, binary relevance, classifier chains, and randomized k-label sets; the crucial features were selected to improve the classification accuracy. Bidgoli et al. [46] used NSGA-III and a binary operator to enhance the exploration of the search process; two objectives (number of features, classification error rate) were optimized simultaneously to maximize the correlation of features or labels. Song et al. [47] proposed a label correlation and group initialization strategy to accelerate the convergence, and two local search operators were guided to enhance the exploitation phase based on the feature redundancy. Kashef et al. [48] mapped the features to a high-dimensional space, and the crucial features were selected by multi-objective optimization with the help of Pareto dominance. Hashemi et al. [49] regarded multi-label feature selection as a bi-objective optimization problem investigating the relevance and redundancy of features and then used Pareto domination to deal with it, differentiating the validity of features as the increase in label dimensions. Paniri et al. [29] proposed a feature selection method based on ant colony optimization that iteratively identifies features to maximize relevance and minimize redundancy using unsupervised and supervised heuristics (MLACO). However, with the increasing number of objectives, it is difficult to capture the non-linear relationship between features and labels with the objective definition based on features. In addition, the evolutionary process easily falls into the local optima, leading to the feature subset still showing redundancy.

### 2.3. Multi-Label Feature Selection Method Based on Graph Structure

The graph structure reveals the underlying relationship by mapping features and labels to nodes and using edges to show the interaction between them. Sun et al. [50] proposed a dual-graph regularization to project features into low-dimensional embeddings, exploring the geometric structure of data, and a sparser weight matrix was computed to preferably accord with the redundant features. Zhang et al. [51] produced pseudo-label matrices by linear regression and label manifolds, and they used dynamic graph Laplacian matrices and feature manifolds to jointly constrain the learning of the feature weight matrix. Fan et al. [52] presented a manifold framework with a regression model to find uncorrelated yet discriminative features, and a low-dimensional representation based on the feature space was utilized to fit the distribution of labels. Hashemi et al. [53] used the correlation distance between features and labels as a matrix, and the PageRank algorithm was applied to rank the importance of each node (or feature) and obtain the optimal feature subset (MGFS). Ma et al. [54] proposed a graph embedding learning framework equipped with adaptive graph diffusion to discover a potential subspace, which preserved the higher-order structural information between samples among four tuples to position crucial features. Hashemi et al. [55] constructed a feature–label graph, where features and labels were used as left and right vertices of a bipartite graph, respectively, and the selected features were ranked according to the weighted correlation distance (BMFS). However, the above methods emphasize the geometric structure while significantly affecting the distribution of features and labels. In addition, the relationship between features and labels is complex in the graph structure, and it makes it difficult to reduce the computational complexity.

## 3. Preliminaries

In this section, the main concepts involved in the proposed method are briefly introduced.

### 3.1. Multi-Label Learning

In multi-label data, each sample contains a feature vector $X_{i}=\left(x_{i1},x_{i2},...x_{id}\right)$ and a label vector $Y_{i}=\left(y_{i1},y_{i2},...,y_{ic}\right)$, where *D* is the number of features and *L* denotes the label dimension. The purpose of multi-label learning is to develop a model of *N* training samples of a dataset, and the labels for new samples are predicted by the model. Multi-label data are depicted in Table 1, where 1 indicates that the sample includes the label and 0 indicates that the sample excludes the label for each row in *Y*.

### 3.2. Mutual Information

In information theory, entropy is a fundamental concept that represents the uncertainty of a random variable. For a discrete random variable *X*, its entropy H(X) [56] is defined as Equation (1).

(1) $$H\left(X\right)=-\sum_{i}P(x_{i})\log P\left(x_{i}\right)$$

where $x_{i}$ is the possible values of *X* and $P(x_{i})$ is the probability of $x_{i}$. Entropy is considered an uncertainty measure, a higher value of entropy indicating more significant uncertainty.

Under the preset of another random variable, the uncertainty of the current variable is defined as the conditional entropy.

(2) $$H\left(X\left|Y\right.\right)=-\sum_{x_{i}\in X}\sum_{y_{i}\in Y}P\left(x_{i},y_{i}\right)\log_{2}\frac{P(x_{i},y_{i})}{P(y_{i})}$$

where $P(x_{i},y_{i})$ is the probability of $x_{i}$ on the condition of Y=y.

Mutual information [57] is calculated based on the difference between the information entropy and the conditional entropy to measure the correlation of two random variables; a higher value indicates a stronger dependency between them, which is defined as Equation (3).

(3) $$I\left(X;Y\right)=H\left(X\right)-H\left(X\left|Y\right.\right)$$

### 3.3. Spectral Clustering

Spectral clustering [58] is an unsupervised learning method based on graph theory, which utilizes the similarity between samples to represent the categorical information. For a given dataset, the similarity matrix *W* between samples is defined as Equation (4) [59].

(4) $$W_{n\times n}=\left\{\begin{array}{c} \exp\left(-\frac{{∥x_{i}-x_{j}∥}_{2}^{2}}{2\sigma^{2}}\right)\;x_{i}\in KNN\left(x_{j}\right)\;\mathrm{or}\;x_{j}\in KNN\left(x_{i}\right)\\ \;\;\;\;\;\;\;\;\;0\;\;\;\mathrm{otherwise} \end{array}\right.$$

(5) $$W_{n\times n}=\frac{W+W^{T}}{2}$$

where *n* denotes the number of samples and $KNN(x_{i})$ represents the neighbor of the *i*-th sample. exp, σ, and *T* denote the exponential function, Gaussian scale function, and transpose operation, respectively. The Laplace matrix [60] is calculated as Equation (6) with *W* and its degree matrix.

(6) L=D−W

The eigenvalue decomposition is conducted for *L*, and the eigenvectors corresponding to the first *k* smallest eigenvalues are selected to generate a new matrix for clustering it.

### 3.4. Non-Dominated Sorting

As for multi-objective optimization, a Pareto front is acted as the solution sets in the space of decision variables, in which it is difficult to further improve one objective function without compromising others. Suppose that there are *M* objective functions, $f_{1}\left(x\right),f_{2}\left(x\right),...,f_{M}\left(x\right)$, aiming to find a group of decision variables, *X*, such that it forms a Pareto front [61] in the solution space, which is defined as Equation (7).

(7) $$ParetoFront=\{x\in X\left|∄\hat{x}\in X,\;\forall i,f_{i}\right.\left(\hat{x}\right)\leq f_{i}\left(x\right),\;\exists j,\;f_{j}\left(\hat{x}\right)<f_{j}\left(x\right)\}$$

To determine the solution that constitutes the Pareto front, a non-dominated sorting [62] is used to identify the dominance relationship between multiple objectives. The solution set is hierarchically ranked to reveal the priority between solutions, thus mining the equilibrium between multiple objectives.

### 3.5. Bipartite Graph Maximum Matching

In a bipartite graph [63,64], the nodes are divided into two vertex-disjoint sets from left to right, which are notated as *U* and *V*. Each edge in the graph connects a vertex from *U* and another from *V*, which is expressed as G=(U,V,E), and *E* is the edge set connecting the nodes on either sides.

**Figure 1:**
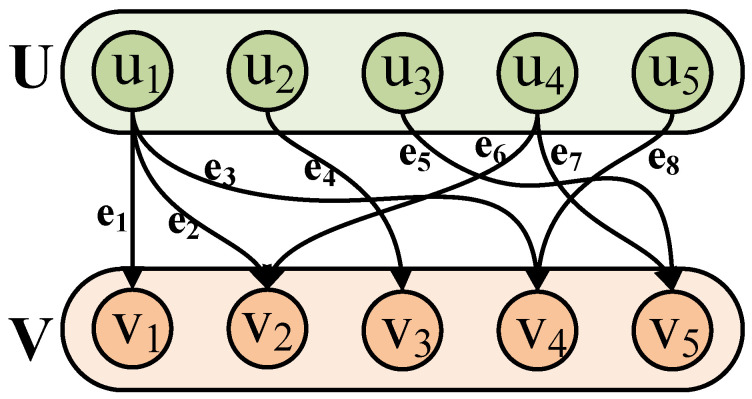
Bipartite graph.

A bipartite graph provides a concise and efficient way to establish the association between two independent entities. In Figure 1, e1–e8 are the matching edges between *U* and *V* [65,66]. To simplify the connectivity relationship between entities, a breadth-first search is used to determine the largest set of nodes in *U* that are paired with a vertex in *V*, and do not overlap with others, making each vertex is matched only once.

## 4. The Proposed Method

In this section, the proposed multi-label feature selection method is presented in the following. The step-by-step procedure of the proposed method is listed in Algorithm 1. First, the shortcomings of previous methods are briefly summarized, and feasible ways are given in the motivation. Further, the key techniques of SAGRL are specificly listed, through the construction of feature–label subgraphs, updating subgraph associations, the integration and deduplication of matched subgraphs, respectively, and obtaining the optimal feature subset.
**Algorithm 1**: SAGRL for multi-label feature selection**Require:** The feature matrix *X* contains *N* samples and *d* features. The label matrix *Y* contains *N* samples and *c* labels. The clustering numbers k1 and k2 are used for the features and labels. **Ensure:**The optimal feature subset *F*.1:FF is calculated by Equation (8) to quantify the correlation of features;2:LL is the frequency of co-occurrence between labels calculated by Equation (9);3:k=k1∗k2;4:F1 is the result by spectral clustering of FF (k1 is the number of clusters);5:L1 is the result by spectral clustering of LL (k2 is the number of clusters);6:*R* is the feature–label similarity matrix calculated by Equation (12);7:Extract submatrix $r\left(i\right)={\left(\begin{array}{ccc} a_{11} & \ldots & a_{1n}\\ \vdots & \ddots & \vdots \\ a_{m1} & \cdots & a_{mn} \end{array}\right)}_{F1(i)*L1(i)}$;8:**for** i=1 to *k* **do**9:    Pf(i) is the non-dominant feature in each subgraph found by Equation (7);10:**end for**11:Updating subgraphs $r^{\prime }\left(i\right)={\left(\begin{array}{ccc} a_{11} & \ldots & a_{1n}\\ \vdots & \ddots & \vdots \\ a_{m1} & \cdots & a_{mn} \end{array}\right)}_{Fp(i)*L1(i)}$;12:**for** i=1 to *k* **do**13:    $F^{\prime }$(i) is the index of the feature combination obtained by one-to-one matching;14:**end for**15:$F^{\prime \prime }$ is obtained by the integration and deduplication of $F^{\prime }$;16:*F* is obtained via ranking by Equation (15);

### 4.1. Motivation

Multi-label data involve feature–feature, label–label, feature–label dependencies, and traditional methods mainly focused on feature redundancy, which fail to fully consider the co-occurrence patterns of labels. In multi-objective optimization, when the number of objectives exceeds a threshold, it may cause most of the features become non-dominated, making the feature subset still redundant. In addition, it is difficult to reflect potential node connections by regarding multi-label data as the graph structure, which affects the relationship between features and their corresponding labels.

For the above problems, SAGRL is utilized for multi-label feature selection to efficiently analyze the features corresponding to each label set. Among them, graph representation learning expresses the complex correlations of features or labels by mapping multi-label data into the graph structure, and the feature and label sets are combined to construct feature–label subgraphs. To better associate the subgraphs, the connection relationship between them is adjusted based on multi-objective optimization, and non-dominated sorting is employed to remove highly relevant features. Features and labels in each subgraph are matched one to one, and all feature combinations are integrated and deduplicated to obtain the optimal feature subset.

### 4.2. Feature–Label Subgraph Construction

**Figure 2:**
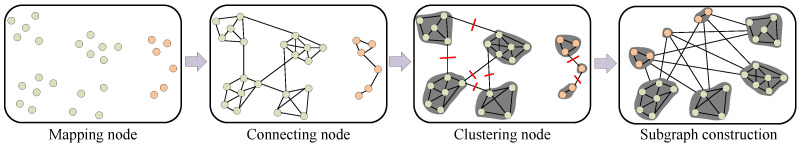
Feature–label subgraph construction.

Multi-label data are viewed as the graph structure, and edges connecting nodes indicate correlation. Spectral clustering is performed from the perspectives of features and labels, the graph is cut to form feature and label sets and then construct feature–label subgraphs. The process of feature–label subgraph construction is shown in Figure 2:

To quantify the correlation of features, mutual information is used as a criterion to capture its linear relationship and also to more effectively measure non-linear feature dependency; the similarity matrix is defined as Equation (8).

(8) $$FC_{d\times d}=\left(\begin{array}{cccc} ff_{11} & ff_{22} & \cdots & ff_{1d}\\ ff_{21} & ff_{22} & \cdots & ff_{2d}\\ \vdots & \vdots & \ddots & \vdots \\ ff_{d1} & ff_{d2} & \cdots & ff_{dd} \end{array}\right)$$

where *d* denotes the number of features, and $F_{ij}$ indicates the correlation of the *i*-th and *j*-th feature that is calculated by Equation (3). The co-occurrence frequency is used to reveal the correlation of labels which visually reflects the probability of the simultaneous occurrence of labels in the dataset. The co-occurrence frequency matrix is defined as Equation (9).

(9) $$LC_{c\times c}=\frac{\sum_{k=1}^{n}\left(y_{k,i}==1\&\&y_{k,j}==1\right)}{n}=\left(\begin{array}{cccc} ll_{11} & ll_{22} & \cdots & ll_{1c}\\ ll_{21} & ll_{22} & \cdots & ll_{2c}\\ \vdots & \vdots & \ddots & \vdots \\ ll_{c1} & ll_{c2} & \cdots & ll_{cc} \end{array}\right)$$

As the connection is established between nodes, two weighted graphs are output and notated as Graph1(F,E1) and Graph2(L,E2). The above graphs are partitioned into $k_{1}$ and $k_{2}$ subgraphs to reduce the space complexity. Taking Graph1 as an example, the minimization of edge weights in the subgraph and the maximization of edge weights between different subgraphs are pursued after partition; for k subgraph nodes $A_{1}$, $A_{2}$, ⋯, $A_{k}$, $A_{i}\cap A_{j}=\emptyset$, $A_{1}\cup A_{2}\cup \cdots \cup A_{k}=V$, the objective function is defined as Equation (10) [67]:

(10) $$\min Cut\left(V\right)=\min\sum_{v_{i}\in A_{k,}v_{j}\in \overset{-}{A_{k}},e_{ij}\in E}w_{ij}$$

where $w_{ij}$ denotes the edge weights between the *i*-th and *j*-th node (feature), min is the minimum, and cut represents cut graph operation. Due to the fuzzy constraints, it is necessary to qualitatively introduce indicator vectors to refine the objective function, and the Lagrange multiplier [68] is used to transform the constrained problem into an unconstrained problem, which is shown in Equation (11).

(11) $$\min Tr(H^{T}LH)\Leftrightarrow \min\lambda$$

where *H* and *L* denote the indicator and Laplace matrices, and λ is the eigenvalue of *L*. The corresponding eigenvectors of λ splice a matrix as an approximate solution of *H*, and the clustering results are achieved by discretization. Feature and label sets are constructed into a subgraph that provides the entity information for considering the relationship between features and labels.

### 4.3. Feature–Label Subgraph Association Updating

**Figure 3:**
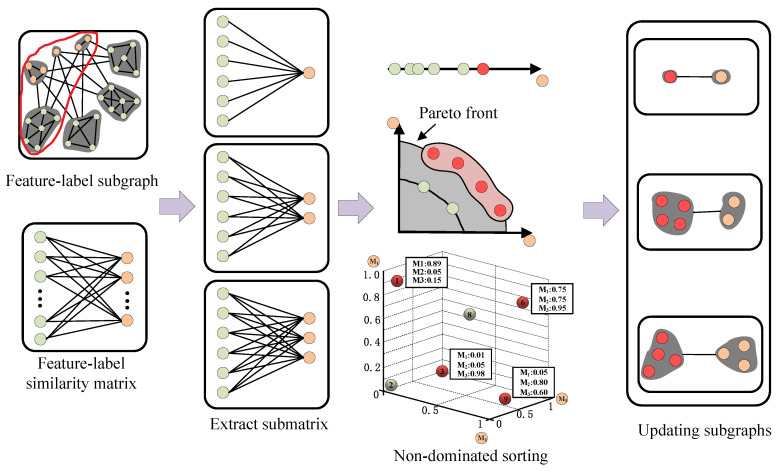
Feature–label subgraph association updating.

The feature–label similarity matrix is calculated to represent the objective value of features on the corresponding labels, and it further extracts the submatrix for each subgraph. Labels are acted as independent objectives, non-dominated sorting is applied to select the features on the Pareto front in each subgraph when multiple objectives are considered at the same time, and the process of feature–label subgraph association adjusting is shown in Figure 3 (Using the subgraph circled with red lines in Figure 2 as examples).

The association degree of features and labels is comprehensively considered by calculating the feature–label similarity matrix, which is defined as Equation (12).

(12) $$R_{d\times c}=1-\frac{\mathrm{cov}(x,y)}{\sqrt{\mathrm{var}(x)\cdot \mathrm{var}(y)}}=\left(\begin{array}{cccc} W_{11} & W_{12} & \cdots & W_{1c}\\ W_{21} & W_{22} & \cdots & W_{21}\\ \vdots & \vdots & \ddots & \vdots \\ W_{d1} & W_{d1} & \cdots & W_{dc} \end{array}\right)$$

where *x* and *y* denote features and labels, respectively, cov(x,y) is the covariance of *x* and *y*, and var(·) indicates the variance.

**Figure 4:**
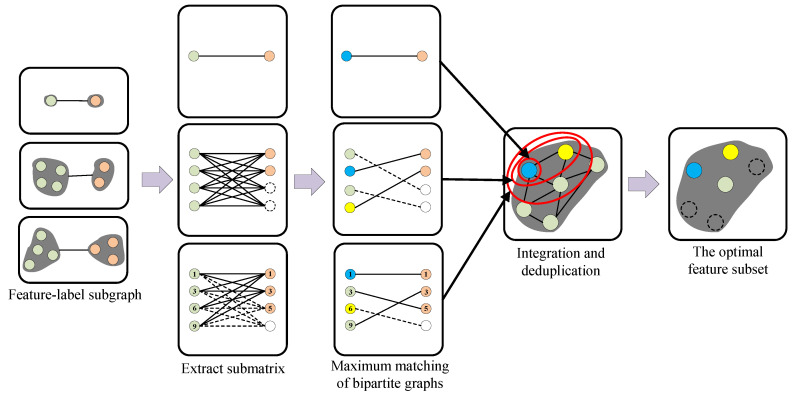
Feature–label subgraph matching.

In multi-objective optimization, the subgraph uses non-dominated sorting to yield its Pareto front, which makes the selected features more fitting to different distributions of labels in each subgraph. The 3rd subgraph in Figure 4 shows 6 features indexed 1, 2, 3, 6, 8, 9 and 3 objectives with labels indexed 1, 3, 5: M1, M2, and M3, and the submatrix is *r*. As for the non-dominated sorting, the red circles 1, 3, 6, and 9 denote the non-dominated features, which are the members of a Pareto optimal set and surround the dominated features (green circles), while the orange circles indicate objectives. Among them, the red circle has a larger *W* value in more than one dimension compared to the others. The feature indexed {1,3,6,9} on the Pareto front is known from the figure, so the feature–label submatrix is updated as $r^{\prime }$. $r={\left(\begin{array}{cccccc} W_{11} & W_{21} & W_{31} & W_{61} & W_{81} & W_{91}\\ W_{13} & W_{23} & W_{33} & W_{63} & W_{83} & W_{93}\\ W_{15} & W_{25} & W_{35} & W_{65} & W_{85} & W_{95} \end{array}\right)}^{T}$$r^{\prime }={\left(\begin{array}{cccc} W_{11} & W_{31} & W_{61} & W_{91}\\ W_{13} & W_{33} & W_{63} & W_{93}\\ W_{15} & W_{35} & W_{65} & W_{95} \end{array}\right)}^{T}$.

### 4.4. Feature–Label Subgraph Matching

Since each subgraph includes multiple objectives, there are still some redundant features. As a result, a bipartite graph is applied to match features and labels by one to one for new subgraphs and acquires a series of feature combinations. In particular, the same features may exist in different subgraphs, avoiding the phenomenon of different labels with same feature combination. The optimal feature subset is obtained by integrating feature combinations after ranking, and the process of feature–label subgraph matching is shown in Figure 4.

Since feature–label subgraph matching requires the number of two sets (left and right vertices) to be equal, a squareization operation is essential on the submatrix. As for the 3rd subgraph in Figure 6 with 4 features (indexed 1, 3, 6, 9) and 3 labels (indexed 1, 3, 5), the submatrix is $r^{\prime }$. The cost matrix is calculated by the all-1 matrix subtracting $r^{\prime }$ and matching the feature–label subgraph to find *B*; the best match scheme is underlined. The feature indexes $F^{\prime }$ are extracted by each label from *B*. As shown in the matrix, 1→1, 3→5, 9→3. The blue and yellow circles (features) in the figure are selected repeatedly by different labels, so it is necessary to conduct integration and deduplication for feature combinations. Additionally, the PageRank [69] algorithm is utilized to assign weights for features, thus ranking the features and obtaining the optimal feature subset, and the weighting of features is defined as shown in Equation (13).

(13) $$PR\left(V_{i}\right)=\left(1-d\right)+d\times \sum_{V_{j}\in In\left(V_{i}\right)}\frac{PR(V_{j})}{L(V_{j})}$$

where $PR(V_{i})$ is the PageRank value of $V_{i}$. *d* is the damping factor, which is usually set to 0.85. $In(V_{i})$ is the nodes directed to $V_{i}$. $L(V_{j})$ is the out-degree of $V_{j}$. 

$\;\;\;\;\;\;r^{\prime }\Rightarrow \left(\begin{array}{ccc} W_{11} & W_{13} & W_{15}\\ W_{31} & W_{33} & W_{35}\\ W_{61} & W_{63} & W_{65}\\ W_{91} & W_{93} & W_{95} \end{array}\right)\Rightarrow \left(\begin{array}{ccc} 0.89 & 0.05 & 0.15\\ 0.01 & 0.05 & 0.98\\ 0.75 & 0.75 & 0.95\\ 0.05 & 0.80 & 0.60 \end{array}\right)\Rightarrow \left(\begin{array}{cccc} 0.89 & 0.05 & 0.15 & 0\\ 0.01 & 0.05 & 0.98 & 0\\ 0.75 & 0.75 & 0.95 & 0\\ 0.05 & 0.80 & 0.60 & 0 \end{array}\right)$

$B^{\prime }\Rightarrow \left(\begin{array}{cccc} \underline{0} & 0.75 & 0.80 & 0\\ 0.91 & 0.78 & \underline{0} & 0.03\\ 0.14 & 0.05 & 0 & \underline{0}\\ 0.84 & \underline{0} & 0.35 & 0 \end{array}\right)$$F^{\prime }\Rightarrow \left(\begin{array}{cccc} \underline{0.89} & 0.05 & 0.15 & 0\\ 0.01 & 0.05 & \underline{0.98} & 0\\ 0.75 & 0.75 & 0.95 & \underline{0}\\ 0.05 & \underline{0.80} & 0.60 & 0 \end{array}\right)$.

## 5. Experimental Studies

In this section, we verify the performance of the proposed method through comprehensive experiments, utilizing datasets in different domains, evaluation metrics, experimental settings, comparisons with other state-of-art multi-label feature selection methods, and the analysis of parameter sensitivity.

### 5.1. Datasets

The datasets used for the experiments are derived from both the Mulan (http://mulan.sourceforge.net/datasets.html, Accessed: 5 August 2024) and Meka (http://waikato.github.io/meka/datasets/, Accessed: 5 August 2024) databases, covering text, image, audio, and bioinformatics domains. Table 2 summarizes the specifications of these datasets, including dataset name (Datasets), number of samples (Samples), number of features (Features), number of labels (Labels), and feature type (Type). In addition, label cardinality (LC) [48] is the cardinality normalized by $\left|L\right|$ defined by Equation (14), label density (LD) [48] is the average number of labels associated with each sample as defined by Equation (15), and the domain of the dataset (Domain) is also included.

(14) $$LC\left(D\right)=\frac{1}{N}\sum_{i=1}^{N}\left|Y_{i}\right|$$

(15) $$LD\left(D\right)=\frac{1}{N}\sum_{i=1}^{N}\frac{\left|Y_{i}\right|}{\left|L\right|}$$

### 5.2. Evaluation Metrics

Among them, Hamming Loss, Ranking Loss, Coverage, Average_Precision, macrof1, and microf1 are used as the evaluation metrics [70] to measure the performance of SAGRL. Let $U=\{\left(x_{i},Y_{i}\right)|1\leq i\leq t\}$ be a test set and $f(x_{i})$ be the predicted label set for unknown instance $x_{i}$.

**Hamming Loss:** This metric evaluates the average error rate over all the binary labels, and ⊕ is the symmetric difference between two sets, where ${\left|\cdot \right|}_{1}$ denotes the $l_{1}$ norm.

(16) $$HL\left(f,u\right)=\frac{1}{t}\sum_{i=1}^{t}\frac{1}{q}{\left|f\left(x_{i}\right)⊕Y_{i}\right|}_{1}$$

**Ranking Loss**: This metric evaluates the fraction of reversely ordered label pairs, $f_{j}\left(x_{i}\right)$ indicates the *j*-th term of $f(x_{i})$, and $\bar{Y_{i}}$ is the complementary set of $Y_{i}$ in *L*.

(17) $$RL\left(f,u\right)=\frac{1}{t}\sum_{i=1}^{t}\frac{\left|\left\{\left(l_{j},l_{k}\right)\left|f_{j}\left(x_{i}\right)\leq f_{k}\left(x_{i}\right),\left(l_{j},l_{k}\right)\in Y_{i}\times \bar{Y_{i}}\right.\right\}\right|}{\left|Y_{i}\right|\left|\bar{Y_{i}}\right|}$$

**Coverage**: This metric evaluates how many steps are needed, on average, to go down the label ranking list so as to cover all the ground-truth labels, and $rank(x_{i},l_{k})$ returns the rank of $l_{k}$ in *L* when all labels are sorted based on f in descending order.

(18) $$CV\left(f,u\right)=\frac{1}{t}\sum_{i=1}^{t}\max_{l_{k\in Y_{i}}}rank\left(x_{i},l_{k}\right)-1$$

**Average_Precision**: This metric evaluates the average fraction of relevant labels ranked higher than a particular label $l_{k}\in Y_{i}$.

(19) $$AP\left(f,u\right)=\frac{1}{t}\sum_{i=1}^{t}\frac{1}{\left|Y_{i}\right|}\sum_{l_{j,}l_{k}\in Y_{i}}\frac{\left|L_{i}=\left\{l_{j}\left|rank\left(x_{i},l_{j}\right)\leq rank\left(x_{i},l_{k}\right)\right.\right\}\right|}{rank(x_{i},l_{k})}$$

**Macrof1**: This metric evaluates the classification accuracy of a label set, which considers F-measure averaging on each label.

(20) $$MaF\left(f,u\right)=\frac{1}{q}\sum_{j=1}^{q}\frac{2\sum_{i=1}^{t}y_{ij}f_{j}\left(x_{i}\right)}{\sum_{i=1}^{t}y_{ij}+\sum_{i=1}^{t}f_{j}\left(x_{i}\right)}$$

**Microf1**: This metric evaluates the classification accuracy of a label set, which considers F-measure averaging on the prediction matrix.

(21) $$MiF\left(f,u\right)=\frac{2{\sum_{i=1}^{t}\left|f\left(x_{i}\right)\cap Y_{i}\right|}_{1}}{\sum_{i=1}^{t}{\left|Y_{i}\right|}_{1}+\sum_{i=1}^{t}{\left|f(x_{i})\right|}_{1}}$$

For Hamming Loss, Ranking Loss, and Coverage, lower values indicate better performance, while for Average_Precision, macrof1, and microf1, higher values indicate better performance.

### 5.3. Experimental Setting

In this section, the proposed method is evaluated on 11 public datasets. To justify the performance, it is compared with several multi-label feature selection methods: PPT-CHI (Pruned Problem Transformation–CHI-square) [38], PPT-MI [37], PMU (Pairwise Multi-Label Utility) [40], D2F [43], MIFS [16], MCLS [17], MDFS [19], MGFS [53], MLACO [29], BMFS [55], and GLFS [18]. All parameters of the comparison methods are set according to the recommendations in the corresponding paper. Among them, 60% of the samples are chosen randomly as the training data and the remaining 40% are used as the test data, and the experimental results are averaged over 20 independent runs. All experiments are performed on a Microsoft Windows 10 operating system Intel(R) Core(TM) i7-10700 CPU using Matlab_R2021b.

MLKNN [71] is a commonly used classifier for multi-label classification tasks that extends the traditional K nearest neighbor classifier. By considering the neighbor distribution of each label, the Bayes rule is applied to predict the applicability of labels.

For the low dimension of features or labels, fewer features are matched to the corresponding labels. For the datasets with less than 300 features, only the evaluation metrics with {1, 5, 10, 15, 20, 25, 30, 35, 40, 45, 50} features are applied for plotting, and for those with more than 300 features, {1, 10, 20, 30, 40, 50, 60, 70, 80, 90, 100} are applied for plotting. In the figure, the horizontal coordinate represents the number of features, and the vertical coordinate represents the evaluation metrics.

### 5.4. Results and Discussion

#### 5.4.1. Results for Different Datasets

**Figure 5:**
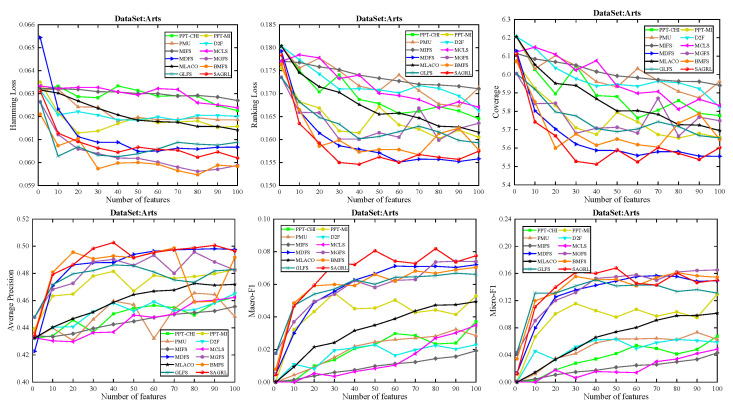
Results on Arts dataset with different numbers of features.

**Figure 6:**
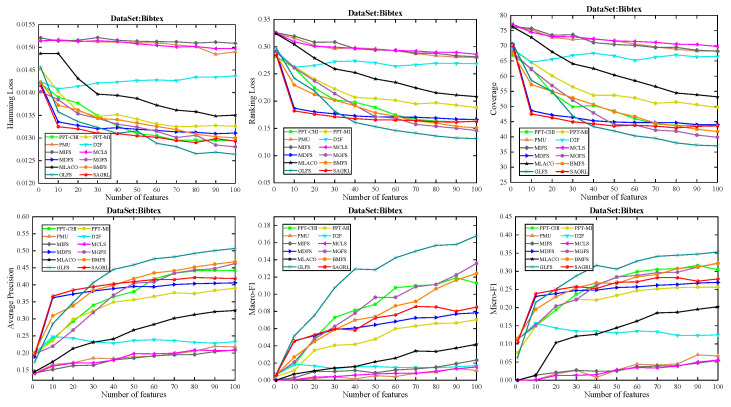
Results on Bibtex dataset with different numbers of features.

**Figure 7:**
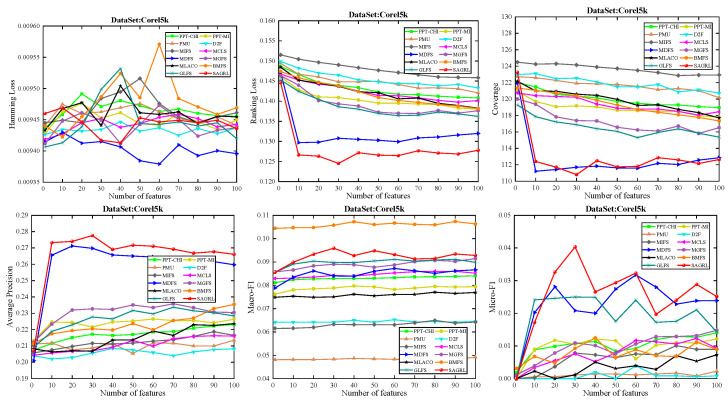
Results on Corel5k dataset with different numbers of features.

**Figure 8:**
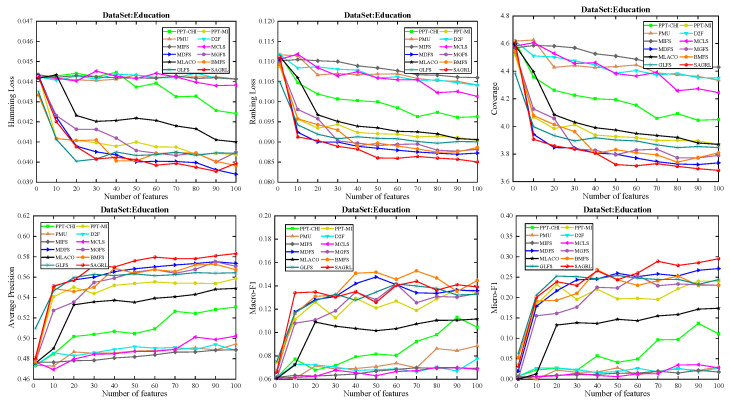
Results on Education dataset with different numbers of features.

**Figure 9:**
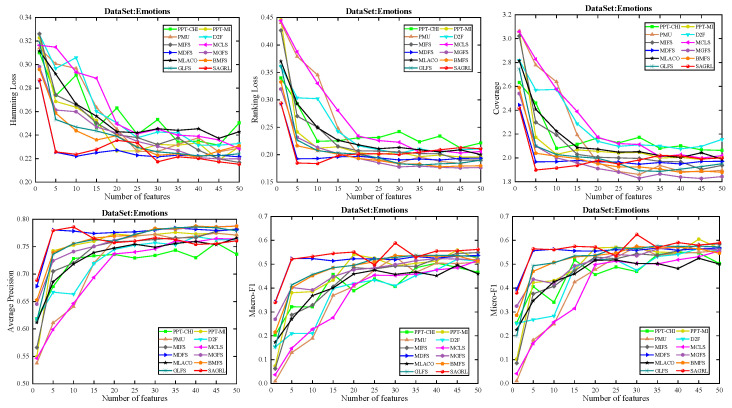
Results on Emotions dataset with different numbers of features.

**Figure 10:**
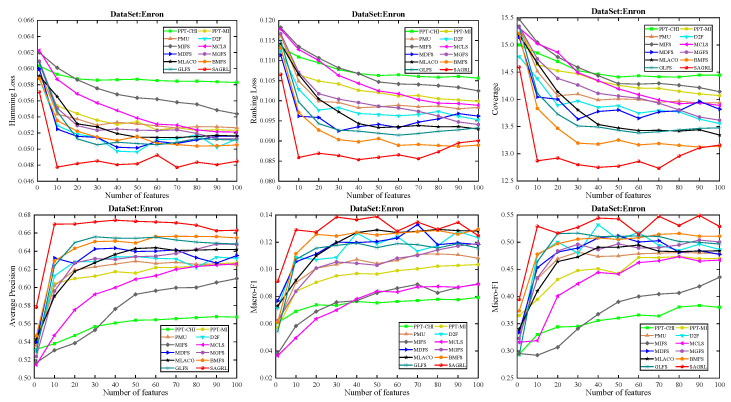
Results on Enron dataset with different numbers of features.

**Figure 11:**
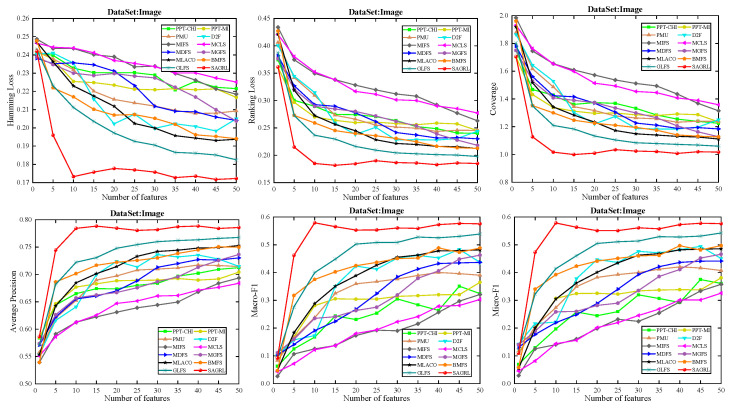
Results on Image dataset with different numbers of features.

**Figure 12:**
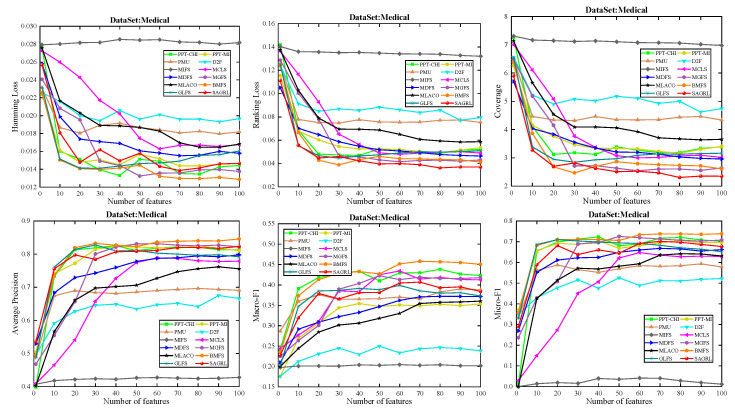
Results on Medical dataset with different numbers of features.

**Figure 13:**
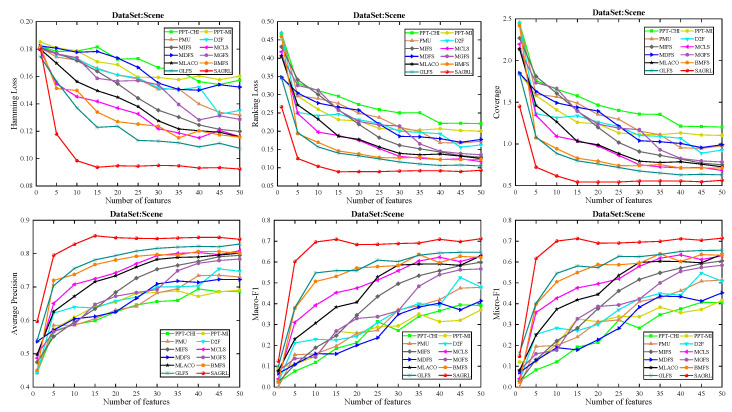
Results on Scene dataset with different numbers of features.

**Figure 14:**
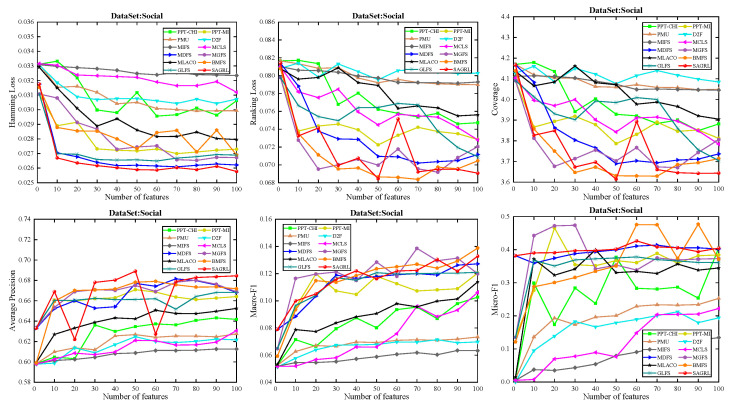
Results on Social dataset with different numbers of features.

**Figure 15:**
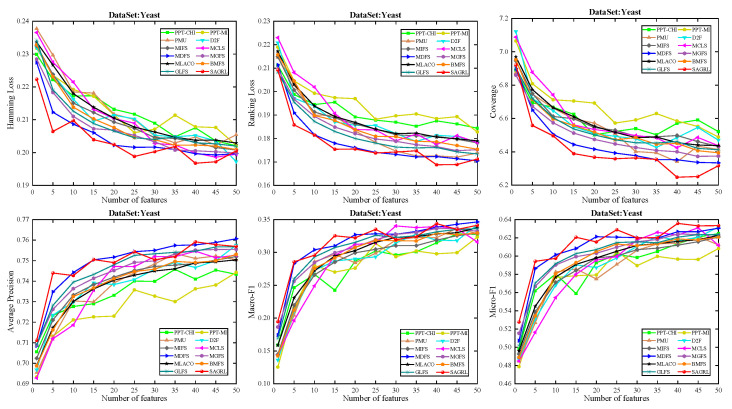
Results on Yeast dataset with different numbers of features.

The curves of comparison methods on 11 datasets in terms of six evaluation metrics are shown in Figure 5, Figure 6, Figure 7, Figure 8, Figure 9, Figure 10, Figure 11, Figure 12, Figure 13, Figure 14 and Figure 15. The experimental results show that the proposed method outperforms most of the comparison methods in six evaluation metrics, which emphasizes the effectiveness of accurately identifying and predicting labels. On the Corel5k dataset, the values of all methods on Hamming Loss are around 0.00945, and the convergence trend is unobvious, which may be related to the inherent characteristics of the dataset as the label dimension is 374 and the LD is only 0.0094. When the number of features is between 50 and 70, the additional features have a certain effect for the ranking on the Social dataset, and the fluctuations in ranking loss, coverage, and average precision are 0.007, 0.27, and 0.06, respectively. On the Bibtex and Education datasets, SAGRL exhibits the same convergence trend as MDFS and the values are better, which performs more prominently when the number of selected features is small and shows the potential to cope with the “curse of dimensionality”. From a domain perspective, the “text” domain demonstrates a clear advantage, particularly on the Bibtex dataset dataset, where performance is superior to other comparative methods when the number of features is kept between 0 and 20. On the Corel5k dataset, while the convergence trend of the Hamming Loss exhibits potential for improvement, other metrics performed well. In contrast, the limited numbers in the “Audio” and “Biology” domains prohibit a more in-depth analysis in these areas.

#### 5.4.2. Comparison of Running Time

Table 3 records the running time (in seconds) of SAGRL with all the comparison methods on 11 datasets, and the experimental results show that the running time of the proposed method is less than 1 s for all datasets. Even on the Bibtex dataset, it takes only 0.641 s, MGFS takes 1.197 s, and the other methods are over 10 s or more. The running time of SAGRL is slower than MGFS and PPT-MI by 0.227 s and 0.094 s on the Corel5k and Medical datasets, respectively, but it outperforms them in terms of classification accuracy, and the running time is obviously excellent on the other datasets. For example, on the Arts, Education, Enron, and Social datasets, SAGRL takes only 0.03 s, 0.042 s, 0.116 s, and 0.115 s, while the others also take 0.14 s, 0.129 s, 0.243 s, and 0.307 s especially for MGFS. SAGRL divides the feature and label sets into smaller, more manageable subgraphs through spectral clustering, minimizing inter-subgraph correlations. Each subgraph is processed individually, reducing computational load. Combined with selective ranking and aggregation mechanisms, it ensures that the computation time stays under 1 s for all datasets. The advantage of running time indicates that SAGRL is fast enough for feature selection and exhibits exceptional consistency and reliability across datasets from different domains.

#### 5.4.3. Statistical Analysis of Evaluation Metrics

To compare the performance of SAGRL with other comparison methods statistically, the Friedman N*N test is used to calculate the ranks on 11 datasets, and the overall wins/ties/losses of SAGRL are summarized versus other methods. A lower sum of ranks indicates better performance against others. In all, the numbers in parentheses are the rankings of methods in different evaluation metrics. The last row denotes the ranking sum. From the rankings shown in Table 4, Table 5, Table 6, Table 7, Table 8, Table 9, Table 10, Table 11, Table 12, Table 13 and Table 14, the proposed method is ranked in the top 3 on the Bibtex, Emotions, and Medical datasets, and it is ranked further on all other datasets are 1st overall. On the Enron, Image, and Scene datasets, it ranks 1st for all evaluation metrics. Table 15 shows the sum of wins/ties/losses for SAGRL versus the others on all datasets. According to the last row, among 66 cases (11 datasets* 6 evaluation metrics), SAGRL significantly wins over PPT-CHI, PPT-MI, PMU, D2F, MIFS, MCLS, MDFS, MGFS, MLACO, BMFS, and GLFS 61, 62, 66, 65, 66, 64, 59, 53, 66, 50, and 54 times, respectively.

### 5.5. Parameter Sensitivity

**Figure 16:**
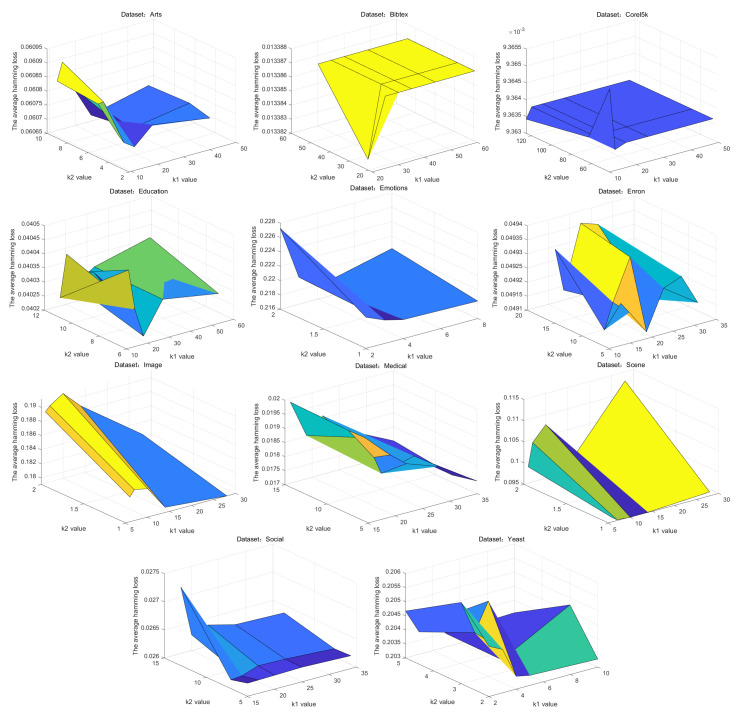
Average Hamming Loss with different pairs of k1 and k2.

The clustering numbers k1 and k2 for features and labels are essential to the classification accuracy. To investigate the influence of clustering number, further experiments are carried out under different values of k1 and k2 combinations as shown in Figure 16. For fewer than 1000 features, k1 is set to F/10, F/20, F/30, F/40, and F/50, respectively. For more than 1000 features, k1 is set to F/30, F/40, F/50, F/60, and F/70. For k2, both are set to L/3, L/6, and L/9 [72]. It is clear from the figure that when F<1000, larger k1 values (e.g., F/10, F/20) have a lower average Hamming Loss. When F>1000 (e.g., Bibtex, Enron, Medical) a smaller k1 (e.g., F/50, F/70) works better. When there are few label dimensions (e.g., Emotions, Image, Scene), larger k2 values (e.g., L/3, L/6) show a more favorable performance. When the are many label dimensions (e.g., Bibtex, Enron), smaller k2 values (e.g., L/6, L/9) tend be advantageous. These results validate that the value of k1 is taken as F/10 for the datasets with fewer features and F/70 for the datasets with high dimensionality. The value of k2 is taken as L/3 for the datasets with fewer labels and L/9 for the datasets with more label dimensions.

## 6. Conclusions

In the paper, a novel SAGRL is proposed for multi-label feature selection. To highlight the correlation of features or labels, feature–label subgraphs are constructed based on spectral clustering, which has not been adequately considered in previous studies based on filter-based methods. For each subgraph combined with graph representation learning, highly relevant features are removed using non-dominated sorting, and the relationship between features and labels is adjusted with the connection of nodes in the subgraph. Further, the augmentation path is located to conduct one-to-one matching based on the bipartite graph. The effectiveness of the proposed method is confirmed by 11 multi-label datasets covering the domains of text, image, audio and biology. Compared with some state-of-art multi-label feature selection methods, our approach shows superior performance both in terms of evaluation metrics and running time. With the uncertainty of category boundaries in multi-label data, there are often more label dimensions than features, which poses a certain challenge regarding accurately selecting the crucial features among them. In the future, it would be useful to extend multi-label feature selection to the multi-modal field by selecting a series of optimal feature subsets that satisfy specific constraints. Moreover, the higher-order correlation of labels is fully considered to guide the model, learn the co-occurrence pattern among various labels, and improve the classification accuracy.

## Figures and Tables

**Table 1:** Multi-label data.

*X*				*Y*			
$X_{1}$	$X_{2}$	**⋯**	$X_{d}$	$Y_{1}$	$Y_{2}$	**⋯**	$Y_{c}$
$x_{11}$	$x_{12}$	⋯	$x_{1d}$	0	1	⋯	0
$x_{21}$	$x_{22}$	⋯	$x_{2d}$	1	0	⋯	0
⋮	⋮	⋱	⋮	⋮	⋮	⋱	⋮
$x_{n1}$	$x_{n2}$	⋯	$x_{nd}$	0	1	⋯	1

**Table 2:** Description of datasets.

Dataset	Samples	Features	Label	Type	LC	LD	Domain
Arts	5000	462	26	Numeric	1.6360	0.0629	Text
Bibtex	7395	1836	159	Nominal	2.402	0.015	Text
Corel5k	5000	499	374	Nominal	3.5220	0.0094	Image
Education	5000	550	33	Numeric	1.4606	0.0443	Text
Emotions	593	72	6	Numeric	1.869	0.311	Audio
Enron	1702	1001	53	Nominal	3.3784	0.0637	Text
Image	2000	294	5	Numeric	1.236	0.247	Image
Medical	978	1449	45	Nominal	1.2454	0.0277	Text
Scene	2407	294	6	Numeric	1.0740	0.1790	Image
Social	5000	1047	39	Numeric	1.2834	0.0329	Text
Yeast	2417	130	14	Numeric	4.237	0.303	Biology

**Table 3:** Running time comparison (in s). N/A denotes that time cost is over 1000 s.

Dataset	SAGRL	PPT-CHI	PPT-MI	PMU	D2F	MIFS	MCLS	MDFS	MGFS	MLACO	BMFS	GLFS
Arts	**0.03**	1.0112	0.3939	274.337	N/A	0.6356	4.196	3.146	0.14	1.749	0.5045	2.762
Bibtex	**0.641**	24.48	23.819	N/A	N/A	16.366	57.092	59.852	1.197	37.508	14.842	137.417
Corel5k	0.496	3.395	3.33	N/A	N/A	15.289	30.2265	34.204	**0.269**	2.042	1.377	10.244
Education	**0.042**	1.198	0.388	405.691	N/A	0.804	9.425	3.293	0.129	3.069	0.776	2.573
Emotions	**0.008**	0.1052	0.008	1.537	15.666	0.038	0.3	0.067	0.033	0.188	0.063	0.411
Enron	**0.116**	0.785	0.645	N/A	N/A	0.789	1.731	1.7	0.243	9.694	2.204	6.798
Image	**0.009**	5.125	0.01	24.733	545.616	0.164	2.186	1.527	0.038	0.899	0.359	1.634
Medical	0.182	0.341	**0.088**	585.991	N/A	0.494	0.791	0.417	0.271	13.364	4.59	1.623
Scene	**0.01**	3.486	0.059	29.156	720.362	0.147	2.799	1.798	0.068	0.945	0.466	2.772
Social	**0.115**	1.849	0.536	931.107	N/A	1.714	13.538	4.019	0.307	10.901	3.45	4.981
Yeast	**0.01**	2.5701	0.0199	17.512	287.1788	0.114	10.512	1.01	0.021	0.242	0.031	1.199

**Table 4:** Average ranking of comparison methods for 6 evaluation metrics by performing the Friedman test on the Arts dataset.

Evaluation Metrics	Method											
	SAGRL	PPT-CHI	PPT-MI	PMU	D2F	MIFS	MCLS	MDFS	MGFS	MLACO	BMFS	GLFS
Hamming Loss	3.64 [4]	10.41 [10]	6.95 [6]	8.09 [9]	7.68 [8]	10.82 [11]	11.09 [12]	5.18 [5]	2.05 [2]	7.14 [7]	**1.45 [1]**	3.50 [3]
Ranking Loss	**2.09 [1]**	8.23 [8]	5.09 [6]	10.41 [11]	10.05 [10]	10.86 [12]	9.50 [9]	2.45 [2]	4.41 [4]	7.23 [7]	3.27 [3]	4.41 [5]
Coverage	**1.73 [1]**	8.14 [8]	4.64 [5]	10.41 [11]	9.95 [10]	10.82 [12]	9.73 [9]	2.36 [2]	4.95 [6]	7.23 [7]	3.73 [3]	4.32 [4]
Average_Precision	**2 [1]**	9.59 [10]	5.27 [6]	9.14 [9]	9 [8]	10.64 [12]	10.27 [11]	3.36 [2]	3.77 [4]	7.27 [7]	3.36 [2]	4.32 [5]
MacroF1	**1.64 [1]**	9.41 [9]	6.09 [6]	8.86 [8]	9.68 [10]	11 [12]	10.64 [11]	3.36 [3]	3.5 [5]	7.14 [7]	3.27 [2]	3.41 [4]
MicroF1	2.64 [3]	9.86 [10]	6.09 [6]	8.59 [8]	8.77 [9]	11 [11]	11.18 [12]	3.64 [4]	**2.41 [1]**	7.5 [7]	2.55 [2]	3.77 [5]
Sum of ranks	**13.74 [1]**	55.64 [10]	34.13 [6]	55.5 [9]	55.13 [8]	65.14 [12]	62.41 [11]	20.35 [3]	21.09 [4]	43.51 [7]	17.63 [2]	23.73 [5]

**Table 5:** Average ranking of comparison methods for 6 evaluation metrics by performing the Friedman test on the Bibtex dataset.

Evaluation Metrics	Method											
	SAGRL	PPT-CHI	PPT-MI	PMU	D2F	MIFS	MCLS	MDFS	MGFS	MLACO	BMFS	GLFS
Hamming Loss	**1.86 [1]**	4.14 [5]	6.73 [7]	10.64 [11]	8.55 [9]	11.82 [12]	10.55 [10]	4.05 [4]	3.64 [3]	8.27 [8]	5.36 [6]	2.41 [2]
Ranking Loss	2.64 [2]	4.82 [6]	6.82 [7]	10.55 [10]	8.55 [9]	11.27 [12]	11.18 [11]	4.18 [5]	3.91 [4]	8.27 [8]	3.45 [3]	**2.36 [1]**
Coverage	2.64 [2]	4.45 [6]	6.73 [7]	10.55 [10]	8.45 [9]	10.82 [11]	11.55 [12]	4.36 [4]	3.36 [3]	8.36 [8]	4.36 [4]	**2.36 [1]**
Average_Precision	3.45 [3]	4.91 [5]	6.36 [7]	10.55 [10]	7.82 [8]	11.55 [12]	10.91 [11]	4.91 [5]	4.73 [4]	8.36 [9]	2.36 [2]	**2.09 [1]**
MacroF1	4.64 [5]	3.23 [3]	7.18 [7]	11.68 [12]	8.41 [8]	9.77 [10]	11.14 [11]	5.27 [6]	3.05 [2]	8.41 [8]	4.23 [4]	**1 [1]**
MicroF1	4.09 [5]	3.64 [3]	6.91 [7]	10.5 [10]	7.91 [8]	10.86 [11]	11.41 [12]	5.09 [6]	4 [4]	8.68 [9]	3.09 [2]	**1.82 [1]**
Sum of ranks	19.32 [2]	25.19 [5]	40.73 [7]	64.47 [10]	49.69 [8]	66.09 [11]	66.74 [12]	27.86 [6]	22.69 [3]	50.35 [9]	22.85 [4]	**12.04 [1]**

**Table 6:** Average ranking of comparison methods for 6 evaluation metrics by performing the Friedman test on the Corel5k dataset.

Evaluation Metrics	Method											
	SAGRL	PPT-CHI	PPT-MI	PMU	D2F	MIFS	MCLS	MDFS	MGFS	MLACO	BMFS	GLFS
Hamming Loss	6.36 [6]	9.73 [11]	6.64 [7]	7.91 [8]	3.55 [2]	8.37 [10]	5.5 [5]	**1.5 [1]**	4.95 [3]	8.18 [9]	9.86 [12]	5.09 [4]
Ranking Loss	**1 [1]**	9 [9]	4.73 [5]	10.09 [10]	10.91 [11]	12 [12]	7.23 [8]	2.36 [2]	4 [4]	6.95 [7]	6.73 [6]	3 [3]
Coverage	2.55 [2]	8.27 [9]	5.36 [5]	10.18 [10]	10.64 [11]	12 [12]	6.27 [6]	**1.73 [1]**	3.73 [4]	8.09 [8]	6.27 [6]	2.91 [3]
Average_Precision	**1.64 [1]**	7.45 [7]	4.73 [5]	9.45 [10]	11.82 [12]	9.09 [9]	10.18 [11]	2.91 [2]	3.18 [3]	8.09 [8]	5 [6]	4.45 [4]
MacroF1	2.14 [2]	6.91 [7]	8 [8]	12 [12]	10.09 [10]	10.91 [11]	5.64 [6]	5.45 [5]	3.73 [4]	9 [9]	**1 [1]**	3.14 [3]
MicroF1	**1.91 [1]**	4.82 [4]	6.09 [6]	10.68 [11]	11.32 [12]	8.55 [9]	7 [8]	2.64 [2]	5 [5]	9.91 [10]	6.91 [7]	3.18 [3]
Sum of ranks	**15.6 [1]**	46.18 [8]	35.55 [5]	60.31 [11]	58.33 [10]	60.92 [12]	41.82 [7]	16.59 [2]	24.59 [4]	50.22 [9]	35.77 [6]	21.77 [3]

**Table 7:** Average ranking of comparison methods for 6 evaluation metrics by performing the Friedman test on the Education dataset.

Evaluation Metrics	Method											
	SAGRL	PPT-CHI	PPT-MI	PMU	D2F	MIFS	MCLS	MDFS	MGFS	MLACO	BMFS	GLFS
Hamming Loss	**2.55 [1]**	9.09 [8]	5.41 [6]	9.68 [11]	9.55 [9]	10.45 [12]	9.64 [10]	3.09 [2]	5.09 [5]	7.09 [7]	3.14 [3]	3.23 [4]
Ranking Loss	**1.73 [1]**	8.18 [8]	5.36 [6]	10.45 [11]	10.27 [10]	11.18 [12]	9.27 [9]	2.36 [2]	4.09 [4]	7.18 [7]	3.73 [3]	4.18 [5]
Coverage	**2 [1]**	8.18 [8]	5.36 [6]	10.27 [11]	10.18 [10]	11.18 [12]	9.55 [9]	2.64 [2]	3.82 [4]	7 [7]	3.64 [3]	4.18 [5]
Average_Precision	**1.36 [1]**	8.36 [8]	5.36 [6]	10.27 [11]	9.73 [9]	11.36 [12]	10.09 [10]	2.45 [2]	4.09 [5]	7.18 [7]	3.91 [4]	3.82 [3]
MacroF1	2.82 [2]	7.95 [8]	4.82 [5]	9.32 [9]	9.68 [10]	10.68 [11]	11.18 [12]	3.18 [3]	5.5 [6]	7.68 [7]	**1.91 [1]**	3.27 [4]
MicroF1	**1.91 [1]**	8.09 [8]	4.73 [5]	9.91 [10]	9.36 [9]	10.73 [11]	10.82 [12]	2.91 [3]	5.59 [6]	7.59 [7]	3.91 [4]	2.45 [2]
Sum of ranks	**12.37 [1]**	49.85 [8]	31.04 [6]	59.9 [10]	58.77 [9]	65.58 [12]	60.55 [11]	16.63 [2]	28.18 [5]	43.72 [7]	20.24 [3]	21.13 [4]

**Table 8:** Average ranking of comparison methods for 6 evaluation metrics by performing the Friedman test on the Emotions dataset.

Evaluation Metrics	Method											
	SAGRL	PPT-CHI	PPT-MI	PMU	D2F	MIFS	MCLS	MDFS	MGFS	MLACO	BMFS	GLFS
Hamming Loss	**2 [1]**	8.95 [9]	6 [6]	7.91 [8]	9.86 [10]	6.95 [7]	10.09 [12]	2.27 [2]	4.86 [5]	10 [11]	4.45 [3]	4.64 [4]
Ranking Loss	6.18 [5]	10 [11]	6.91 [7]	6.55 [6]	9.09 [9]	7.27 [8]	10.45 [12]	3.64 [3]	**2.36 [1]**	9.09 [9]	2.55 [2]	3.91 [4]
Coverage	4.91 [5]	10 [10]	7.68 [8]	5.82 [6]	10.55 [11]	6.45 [7]	10.55 [11]	4.18 [3]	**1.73 [1]**	8.41 [9]	3.09 [2]	4.64 [4]
Average_Precision	6.45 [6]	10.18 [11]	5.45 [5]	7.82 [8]	9.36 [10]	6.73 [7]	10.45 [12]	**2.36 [1]**	3.73 [3]	9.09 [9]	2.64 [2]	3.73 [3]
MacroF1	**1.91 [1]**	9.09 [8]	5.18 [5]	9.64 [10]	10 [11]	6.64 [7]	10.45 [12]	2.91 [2]	5.45 [6]	9.09 [8]	4.09 [4]	3.55 [3]
MicroF1	**1.91 [1]**	8.82 [9]	5 [5]	9.18 [10]	8.55 [8]	6.82 [7]	10.82 [12]	3.27 [2]	5.82 [6]	9.73 [11]	4.45 [4]	3.64 [3]
Sum of ranks	23.36 [3]	57.04 [10]	36.22 [6]	46.92 [8]	57.41 [11]	40.86 [7]	62.81 [12]	**18.63 [1]**	23.95 [4]	55.41 [9]	21.27 [2]	24.11 [5]

**Table 9:** Average ranking of comparison methods for 6 evaluation metrics by performing the Friedman test on the Enron dataset.

Evaluation Metrics	Method											
	SAGRL	PPT-CHI	PPT-MI	PMU	D2F	MIFS	MCLS	MDFS	MGFS	MLACO	BMFS	GLFS
Hamming Loss	**1 [1]**	11.55 [12]	8.18 [8]	8.27 [9]	3.68 [3]	11.09 [11]	9.73 [10]	3.91 [4]	6.73 [7]	6.36 [6]	3.5 [2]	4 [5]
Ranking Loss	**1.18 [1]**	10.91 [11]	9.09 [9]	7.45 [8]	5.64 [6]	11.36 [12]	9.91 [10]	4.36 [4]	7.36 [7]	5 [5]	2.09 [2]	3.64 [3]
Coverage	**1.09 [1]**	10.73 [11]	9.45 [10]	7.55 [7]	5.09 [5]	11.27 [12]	9.09 [9]	5.27 [6]	7.82 [8]	4.55 [4]	2.27 [2]	3.82 [3]
Average_Precision	**1 [1]**	11.27 [11]	8 [9]	7.82 [8]	6.55 [7]	11.27 [11]	10 [10]	4.91 [4]	6 [6]	5.45 [5]	2.45 [2]	3.27 [3]
MacroF1	**1.18 [1]**	11.18 [12]	8.91 [9]	7.55 [8]	4.55 [5]	10.64 [10]	11 [11]	4.09 [4]	7.09 [7]	3.09 [3]	2.91 [2]	5.82 [6]
MicroF1	**1.09 [1]**	11.55 [12]	8.45 [9]	7.36 [8]	4.45 [4]	11.18 [11]	9.91 [10]	5.18 [5]	5.27 [6]	6.64 [7]	3 [2]	3.91 [3]
Sum of ranks	**6.54 [1]**	67.19 [12]	52.08 [9]	46 [8]	29.96 [5]	66.81 [11]	59.64 [10]	27.72 [4]	40.27 [7]	31.09 [6]	16.22 [2]	24.46 [3]

**Table 10:** Average ranking of comparison methods for 6 evaluation metrics by performing the Friedman test on the Image dataset.

Evaluation Metrics	Method											
	SAGRL	PPT-CHI	PPT-MI	PMU	D2F	MIFS	MCLS	MDFS	MGFS	MLACO	BMFS	GLFS
Hamming Loss	**1.45 [1]**	9 [10]	7.55 [9]	6.82 [6]	5.36 [5]	11 [11]	11.55 [12]	6.82 [6]	7 [8]	4.45 [4]	4.36 [3]	2.64 [2]
Ranking Loss	**1.09 [1]**	7.36 [8]	6.82 [6]	7.91 [10]	6.18 [5]	11.64 [12]	11.18 [11]	7.18 [7]	7.45 [9]	4.45 [4]	4.36 [3]	2.36 [2]
Coverage	**1.09 [1]**	7.95 [10]	6.64 [6]	7.82 [9]	6.45 [5]	11.73 [12]	11.18 [11]	6.91 [7]	7.14 [8]	4.27 [3]	4.45 [4]	2.36 [2]
Average_Precision	**1 [1]**	7.64 [9]	7.27 [7]	7.27 [7]	6.18 [5]	11.64 [12]	11.18 [11]	7.18 [6]	7.73 [10]	4.18 [3]	4.36 [4]	2.36 [2]
MacroF1	**1.45 [1]**	9.64 [10]	7.45 [9]	6.82 [6]	5.05 [5]	11.55 [12]	11.36 [11]	6.91 [7]	6.91 [7]	4.41 [4]	4.09 [3]	2.36 [2]
MicroF1	**1.45 [1]**	9.82 [10]	7.64 [9]	6.55 [6]	3.91 [3]	11.45 [12]	11.36 [11]	7.18 [8]	7.09 [7]	5.18 [5]	4.09 [4]	2.27 [2]
Sum of ranks	**7.53 [1]**	51.41 [10]	43.37 [9]	43.19 [7]	33.13 [5]	69.01 [12]	67.81 [11]	42.18 [6]	43.32 [8]	26.94 [4]	25.71 [3]	14.35 [2]

**Table 11:** Average ranking of comparison methods for 6 evaluation metrics by performing the Friedman test on the Medical dataset.

Evaluation Metrics	Method											
	SAGRL	PPT-CHI	PPT-MI	PMU	D2F	MIFS	MCLS	MDFS	MGFS	MLACO	BMFS	GLFS
Hamming Loss	4.91 [6]	3.64 [2]	4.36 [5]	8.55 [8]	9.73 [11]	12 [12]	9.27 [10]	6.91 [7]	3.91 [3]	9 [9]	**1.45 [1]**	4.27 [4]
Ranking Loss	**1.45 [1]**	6.09 [6]	6.36 [7]	9.27 [10]	10 [11]	11.91 [12]	7.27 [8]	5.27 [5]	4.09 [3]	9.18 [9]	2.55 [2]	4.55 [4]
Coverage	**1.36 [1]**	6.82 [7]	6.45 [6]	9.18 [9]	10.36 [11]	12 [12]	6.82 [7]	4.91 [4]	3.45 [3]	9.18 [9]	2.55 [2]	4.91 [4]
Average_Precision	4 [3]	4.55 [5]	3.64 [2]	9 [9]	10.09 [11]	11.82 [12]	8.73 [8]	6.36 [7]	4.45 [4]	9 [9]	**1.64 [1]**	4.73 [6]
MacroF1	5.27 [5]	2.09 [2]	9.18 [9]	5.64 [7]	11 [11]	11.82 [12]	4 [3]	7.82 [8]	4.55 [4]	9.55 [10]	**1.55 [1]**	5.55 [6]
MicroF1	5.18 [6]	3.36 [2]	4 [3]	8.64 [8]	9.82 [11]	11.95 [12]	9.36 [10]	6.73 [7]	4.45 [5]	8.77 [9]	**1.45 [1]**	4.27 [4]
Sum of ranks	22.17 [2]	26.55 [4]	33.99 [6]	50.28 [9]	61 [11]	71.5 [12]	45.45 [8]	38 [7]	24.9 [3]	54.68 [10]	**11.19 [1]**	28.28 [5]

**Table 12:** Average ranking of comparison methods for 6 evaluation metrics by performing the Friedman test on the Scene dataset.

Evaluation Metrics	Method											
	SAGRL	PPT-CHI	PPT-MI	PMU	D2F	MIFS	MCLS	MDFS	MGFS	MLACO	BMFS	GLFS
Hamming Loss	**1.36 [1]**	11.18 [11]	11.27 [12]	7.82 [8]	8.55 [9]	6.45 [6]	3.64 [3]	10.27 [10]	7.05 [7]	4.64 [5]	3.68 [4]	2.09 [2]
Ranking Loss	**1 [1]**	11.68 [12]	8.27 [9]	9.73 [11]	8.23 [8]	7.36 [6]	4.09 [4]	8.64 [10]	8.14 [7]	5.05 [5]	3.73 [3]	2.09 [2]
Coverage	**1 [1]**	11.68 [12]	8.27 [9]	9.73 [11]	8.14 [7]	7.36 [6]	4.18 [4]	8.73 [10]	8.14 [7]	5.05 [5]	3.64 [3]	2.09 [2]
Average_Precision	**1 [1]**	11.23 [12]	9.00 [10]	10.09 [11]	8.23 [8]	7.27 [8]	4.09 [6]	8.73 [4]	7.59 [9]	5.05 [7]	3.64 [5]	2.09 [3]
MacroF1	**1 [1]**	10.86 [12]	9.73 [10]	9 [9]	8.32 [8]	6.91 [6]	4.36 [4]	9.91 [11]	7.32 [7]	4.5 [5]	3.91 [3]	2.18 [2]
MicroF1	**1.09 [1]**	11.32 [12]	9.36 [10]	9.18 [9]	7.95 [8]	7 [6]	4.45 [4]	10 [11]	7.14 [7]	4.59 [5]	3.82 [3]	2.09 [2]
Sum of ranks	**7 [1]**	67.86 [12]	55.9 [10]	55.28 [9]	49.24 [8]	41.9 [6]	25.27 [4]	56.1 [11]	45.11 [7]	28.51 [5]	22.69 [3]	13.18 [2]

**Table 13:** Average ranking of comparison methods for 6 evaluation metrics by performing the Friedman test on the Social dataset.

Evaluation Metrics	Method											
	SAGRL	PPT-CHI	PPT-MI	PMU	D2F	MIFS	MCLS	MDFS	MGFS	MLACO	BMFS	GLFS
Hamming Loss	**1.41 [1]**	9.14 [9]	4.55 [5]	8.86 [8]	9.36 [10]	11.55 [12]	11.09 [11]	2.41 [2]	4.27 [4]	6.73 [7]	5.45 [6]	3.18 [3]
Ranking Loss	2.95 [3]	8.59 [9]	4.82 [5]	10.45 [11]	11 [12]	10.23 [10]	6.64 [7]	4.41 [4]	2.55 [2]	8.32 [8]	**2.05 [1]**	6 [6]
Coverage	2.68 [2]	8.41 [8]	5.18 [5]	10.32 [11]	11 [12]	9.73 [10]	6.09 [7]	4.5 [4]	2.91 [3]	8.82 [9]	**2.45 [1]**	5.91 [6]
Average_Precision	**2.59 [1]**	8.32 [8]	4.55 [6]	8.86 [9]	10.45 [10]	11.73 [12]	10.55 [11]	3.95 [4]	2.82 [2]	6.73 [7]	3.09 [3]	4.36 [5]
MacroF1	2.77 [2]	8.41 [8]	4.91 [6]	9.5 [9]	10.45 [11]	11.73 [12]	9.82 [10]	3.91 [5]	**2.64 [1]**	7.09 [7]	2.91 [3]	3.86 [4]
MicroF1	**2.05 [1]**	6.86 [8]	4.73 [4]	8.95 [9]	10.36 [10]	11.73 [12]	10.82 [11]	2.77 [2]	4 [3]	6.09 [7]	4.82 [5]	4.82 [5]
Sum of ranks	**14.45 [1]**	49.73 [8]	28.74 [6]	56.94 [10]	62.62 [11]	66.7 [12]	55.01 [9]	21.95 [4]	19.19 [2]	43.78 [7]	20.77 [3]	28.13 [5]

**Table 14:** Average ranking of comparison methods for 6 evaluation metrics by performing the Friedman test on the Yeast dataset.

Evaluation Metrics	Method											
	SAGRL	PPT-CHI	PPT-MI	PMU	D2F	MIFS	MCLS	MDFS	MGFS	MLACO	BMFS	GLFS
Hamming Loss	**1.73 [1]**	9.45 [10]	9.64 [11]	9.91 [12]	7.82 [8]	6.73 [7]	6.64 [6]	2 [2]	3.45 [3]	9.18 [9]	5.55 [4]	5.91 [5]
Ranking Loss	**1.45 [1]**	9.91 [11]	11.55 [12]	4.82 [5]	8.18 [8]	7.73 [7]	9 [9]	2 [2]	4.45 [4]	9 [9]	6.73 [6]	3.18 [3]
Coverage	**1.55 [1]**	9.09 [11]	11.45 [12]	5.82 [6]	7.5 [7]	8.64 [8]	9 [10]	2.18 [2]	3.09 [3]	8.64 [8]	5.73 [5]	5.32 [4]
Average_Precision	2.18 [2]	10 [11]	11.55 [12]	7.64 [8]	7.73 [9]	6.91 [5]	7.32 [6]	**1.55 [1]**	4.09 [4]	8.73 [10]	7.36 [7]	2.95 [3]
MacroF1	2.73 [2]	9.45 [11]	10.45 [12]	7.55 [7]	8.64 [9]	9.09 [10]	6.27 [5]	**1.82 [1]**	4 [4]	6.45 [6]	7.64 [8]	3.91 [3]
MicroF1	**1.45 [1]**	8 [9]	10.73 [12]	9.27 [11]	7.73 [8]	8.91 [10]	7.55 [7]	2.18 [2]	4.18 [4]	6.91 [5]	7 [6]	4.09 [3]
Sum of ranks	**11.09 [1]**	55.9 [11]	65.37 [12]	45.01 [6]	47.6 [8]	48.01 [9]	45.78 [7]	11.73 [2]	23.26 [3]	48.91 [10]	40.01 [5]	25.36 [4]

**Table 15:** Results of win/tie/loss for SAGRL versus other methods on 6 evaluation metrics.

Evaluation Metrics	SAGRL Against											
	PPT-CHI	PPT-MI	PMU	D2F	MIFS	MCLS	MDFS	MGFS	MLACO	BMFS	GLFS	GLFS
Hamming Loss	10/0/1	10/0/1	11/0/0	10/0/1	11/0/0	10/0/1	10/0/1	8/0/3	11/0/0	9/0/2	8/0/3	5.91 [5]
Ranking Loss	11/0/0	11/0/0	11/0/0	11/0/0	11/0/0	11/0/0	10/0/1	9/0/2	11/0/0	9/0/2	9/0/2	3.18 [3]
Coverage	11/0/0	11/0/0	11/0/0	11/0/0	11/0/0	11/0/0	9/0/2	10/0/1	11/0/0	9/0/2	9/0/2	5.32 [4]
Average_Precision	11/0/0	9/0/2	11/0/0	11/0/0	11/0/0	11/0/0	9/0/2	10/0/1	11/0/0	8/0/3	9/0/2	2.95 [3]
MacroF1	9/0/2	11/0/0	11/0/0	11/0/0	11/0/0	10/0/1	10/0/1	8/0/3	11/0/0	7/0/4	10/0/1	3.91 [3]
MicroF1	9/0/2	10/0/1	11/0/0	11/0/0	11/0/0	11/0/0	11/0/0	8/0/3	11/0/0	8/0/3	9/0/2	4.09 [3]
In total	61/0/5	62/0/4	66/0/0	65/0/1	66/0/0	64/0/2	59/0/7	53/0/13	66/0/0	50/0/16	54/0/12	25.36 [4]

## Data Availability

Data are contained within the article.
